# Control of Stimulated Emission of Tin Perovskites through Polymeric Diffractive Gratings

**DOI:** 10.1021/acsphotonics.5c00471

**Published:** 2025-05-21

**Authors:** Juan P. Martínez-Pastor, Jesús Sánchez-Díaz, José M. Villalvilla, Sandra Soriano-Díaz, José A. Quintana, Iván Mora-Seró, María A. Díaz-García, Isaac Suárez

**Affiliations:** † UMDO, Instituto de Ciencia de los Materiales, 16781 Universidad de Valencia, Valencia 46980, Spain; ‡ Institute of Advanced Materials (INAM), 16748 Universitat Jaume I, Castelló de la Plana, Castelló 12006, Spain; § Departamento de Física Aplicada and Instituto Universitario de Materiales de Alicante (IUMA), Universidad de Alicante, Alicante 03080, Spain; ∥ Departamento de Ingeniería Electrónica, Escuela Técnica Superior de Ingeniería, Universidad de Valencia, Valencia 46100, Spain; ⊥ Departamento de Óptica, Farmacología y Anatomía and IUMA, Universidad de Alicante, Alicante 03080, Spain

**Keywords:** FASnI_3_, random lasing, amplified spontaneous emission, DFB, holographic lithography

## Abstract

FASnI_3_ (FA: formamidinium) polycrystalline perovskite thin films have demonstrated to be an excellent gain media and less toxic alternative to the Pb-containing perovskite light emitters. However, the instability of Sn perovskites prevents the postprocessing of the film after deposition, making its integration in two-dimensional optical architectures challenging. In this article, FASnI_3_ polycrystalline thin films are successfully integrated with polymeric diffractive gratings under cost-effective and industrial compatible technology. The gratings are fabricated under holographic techniques, allowing patterning on large areas (≈1 cm^2^) and the posterior deposition of the perovskite films or cladding layers. The grating period demonstrated to be an adequate tuning parameter to control the amplified spontaneous emission (ASE) properties. Notably, the overlap of the mode with the emission band represents a suitable mechanism to selectively enhance the generation of the ASE (tuned device) above a moderate threshold of 100 μJ/cm^2^ and extract the random lasing lines created on the grains of the film (detuned device). These results represent a significant advance on Sn-perovskite-based optical sources, offering cost-effective technology for next-generation photonic applications.

## Graphical abstract

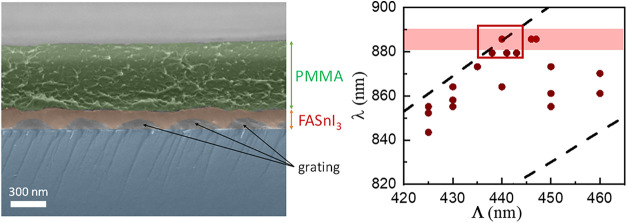

## Introduction

Lead free perovskites (LFPs) have recently emerged as promising materials for photonics and photovoltaics.^1–3^ Particularly, the substitution of Pb with divalent Sn in the perovskite formula ABX_3_ (A is an organic/inorganic cation, X is a halide anion, X = Cl, Br, I, B = Pb or Sn) represents the most straightforward and successful approach to develop low-cost semiconductors with excellent optoelectronic properties and reduced toxicity.^4,5^ Although ASnX_3_ perovskites are inherently deteriorated under ambient conditions (oxygen, moisture, and light), the use of antioxidants (to avoid Sn^2+^ to Sn^4+^ oxidation), surface passivation, or protective layers has been proven successful to mitigate degradation.^6^ Nowadays, ASnX_3_ LFPs are successfully deposited on polycrystalline thin films, demonstrating reasonably stable operation in photodetection,^7,8^ photoconversion,^9,10^ or light emission functionalities.^11,12^

Similarly to Pb-containing perovskite counterparts, ASnX_3_ LFPs present ideal features to be an excellent gain medium, such as high light absorption above the band gap,^13^ long radiative lifetimes,^14,15^ or tunable band gap with the composition^16^ Moreover, the photoluminescence (PL) band in these compounds is shifted to the 850–900 nm window,^15–17^ which represents an advantage compared to Pb perovskites, which are unable to produce lasing above 800 nm. Notably, ASnI_3_ polycrystalline films synthesized with methylammonium (MA), formamidinium (FA), and cesium (Cs) cations have demonstrated the ability to generate amplified spontaneous emission (ASE) and/or random lasing (RL).^15–18^ Particularly promising are the results obtained with FASnI_3_ thin films, for which some of the authors developed the appropriate technological methods to deposit polycrystalline layers with excellent efficiency of emission and improved stability.^10^ These films demonstrated high enough optical gain coefficients, 2000 cm^–1^, to generate ASE under very low thresholds, 100 nJ/cm^2^ and 1 μJ/cm^2^ in rigid and flexible waveguides, respectively.^19^ More interestingly, the films exhibited spectrally reproducible and narrow RL lines on the ASE band,^18,19^ which represents an interesting alternative to implement eco-friendly and cost-effective optical sources. At this stage, the integration of the FASnI_3_ thin films into an optical architecture is the next step toward the development of LFP-integrated optical technology and to further improve the emission properties of these materials. Indeed, a resonator represents a suitable strategy to enhance the radiative recombination rate,^20^ reduce the threshold,^21^ and/or to control the emission properties.^22^ In this sense, we have recently demonstrated that the spectral overlap of the resonant mode in a vertical cavity with the PL band of FASnI_3_ films can enhance the RL properties toward single-mode emission with a quality factor (*Q*) as high as ∼10^3^ at room temperature.^23^ Nevertheless, apart from this work, the integration of ASnI_3_ into optical resonators is elusive and further investigations become necessary toward the development of an LFP photonic technology.

Among the different optical architectures available to demonstrate lasing, a distributed feedback (DFB) device meets the requirements of an industrial manufacturing with optimum performances.^24^ This structure consists of an interference grating etched on the active medium or its adjacent layers.^25^ The fact that the refractive index is periodically alternated on the waveguide structure results in one resonant wavelength that accomplishes the Bragg condition, i.e., single-mode emission. Nowadays, DFB gratings can be fabricated under imprint or stamp templates,^26^ which are compatible with the scalable technology and are useful for perovskite materials, because these techniques prevent the use of undesirable resists or solvents. In this way, DFB gratings have been successfully integrated with Pb perovskite films and demonstrated single-mode lasers with femtosecond^27,28^ and nanosecond^29–31^ pulsed excitations and even under continuous wave operation.^32^ In these works, the DFBs were fabricated by either etching the perovskite film with a stamp or by patterning a cladding layer deposited on the semiconductor. In both cases, the corrugation presents a high enough index contrast to boost the quality factor up to 10^4^, which results in relatively low thresholds (≈1 μJ/cm^2^). In return, the geometrical parameters of the structure needed to be precisely adjusted to match the resonant wavelength with the emission band of the semiconductor, which reduced the flexibility of the fabrication procedure. More importantly, the inherent stability of Sn perovskite prevents the postprocessing of the film after deposition, which is required in the aforementioned methods to fabricate the grating, hence making the demonstration of DFB devices more challenging. To the best of our knowledge, there is only one publication where a two-dimensional PEA_2_SnI_4_ thin film is deposited on a DFB resonator to enhance the generation of ASE at 77 K.^33^

In this work, we successfully demonstrate the tailoring of the ASE and RL emissions of FASnI_3_ polycrystalline thin films at room temperature through the use of a polymeric grating layer. The device is based on a water-soluble dichromate gelatin (DCG) photoresist layer with an engraved one-dimensional grating fabricated by holographic lithography (HL) and subsequent dry etching and covered by a FASnI_3_ film. This grating fabrication method not only avoids the postprocessing of the semiconductor film but is also useful for industrial manufacturing as it allows the patterning on large areas (≈1 cm^2^) and the posterior layer deposition.^34–36^ Besides, this technique enables a straightforward adjustment of the grating period (Λ),^34^ which is used here as a varying parameter to tune the resonant wavelength of the cavity (λ_0_) over the PL band of the semiconductor. This flexibility is convenient for FASnI_3_ semiconductors, where the RL generated within the film competes with the vertical resonant mode of the cavity, resulting in a narrow range of optimal grating periods for proper operation.^31^ A resonant device is found for Λ = 440 nm, where λ_0_ overlaps the ASE band. Thus, the cavity mode dominates over the RL, and the ASE band becomes enhanced above a moderate threshold of 100 μJ/cm^2^. Conversely, a detuned device overlapping the exciton absorption resonance serves to efficiently extract the RL light. These results can lead to important implications in the Sn-perovskite community toward the development of cheap optical sources.

## Device Design and Fabrication

The fabricated gratings were designed to provide feedback in the second-order diffraction mode (*m* = 2) in the Bragg equation

1 $$m\cdot \lambda_{0}=2\cdot n_{\mathrm{eff}}\cdot \Lambda$$

where λ_0_ is the wavelength that gets reinforced along the waveguide, *m* is the diffraction order, Λ is the grating period, and *n*_eff_ is the effective refractive index of the waveguide mode. For such structures, emission occurs preferentially in a direction perpendicular to the sample, as illustrated in Figure 1a. Several devices (see Table S1, Supporting Information) were prepared using the scheme shown in Figure 1a and following the procedure described in the Experimental Section. Initially, a 110 nm thick DCG film is deposited on a fused silica (FS) substrate, and a one-dimensional grating is engraved with the desired period (Λ) by HL and subsequent dry etching (under oxygen). Compared to other nanolithography techniques, HL is a cost-effective method that enables the patterning of low grating periods (down to 200 nm for DCG)^37^ over large areas (1 cm^2^).^34–36^ Besides, HL offers significant advantages to optimize DFB devices,^37,38^ as it enables straightforward and precise adjustment of Λ and facilitates the creation of gratings with different periodicities in the same fabrication run. In this way, we prepared a set of samples where Λ ranged from Λ = 425 nm to Λ = 460 nm (see Table S1). Then, a FASnI_3_ film was spin-coated over the DCG grating and capped with a ≈0.5 μm thick film of poly­(methyl methacrylate) (PMMA) as a protective layer. The thickness of FASnI_3_ was optimized to 200 nm in our previous publications, where we demonstrated an efficient generation of ASE in both waveguide and backscattering geometries.^18,19^ Indeed, the FASnI_3_ films exhibit high crystallinity and uniformity, as shown in the X-ray diffraction (XRD) pattern in Figure S1 (Supporting Information), along with excellent homogeneity and minimal surface roughness (<2.6 nm), as evidenced by the top-view scanning electron microscope (SEM) image and atomic force microscopy (AFM) analysis in Figure S2 (Supporting Information) and Figure 1b, respectively. A representative SEM cross-sectional image of one of the devices (with Λ = 440 nm) is shown in Figure 1c. Besides, a SEM cross-sectional image of the device without the grating layer is presented in Figure 1d.

**Figure 1:**
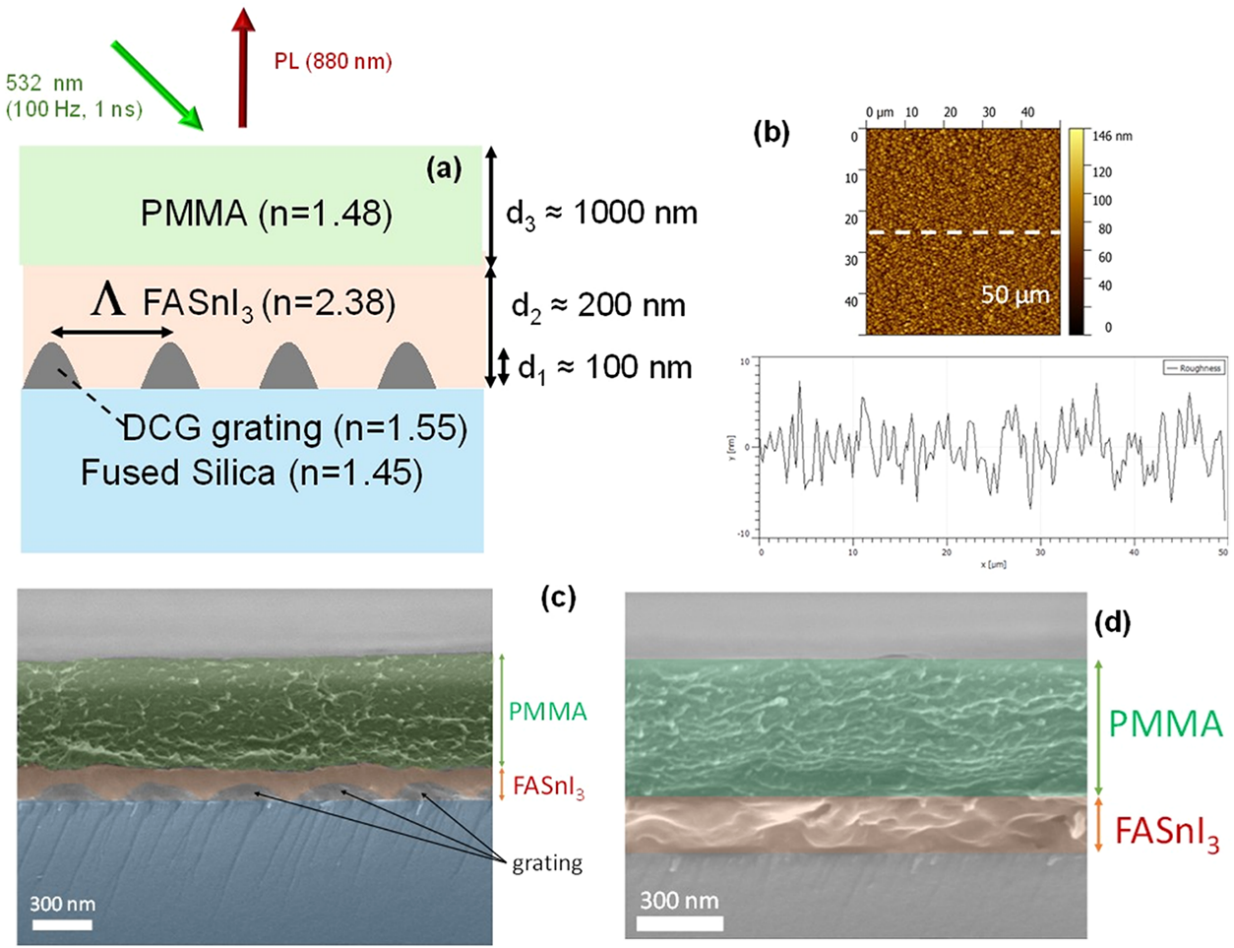
(a) Structure of the device used in this work and experimental geometry for excitation and collection. (b) AFM image of the FASnI_3_ film. (c) SEM cross section of the device with a grating with Λ = 440 nm. (d) SEM cross section of the device without grating.

The geometrical parameters (thickness and refractive index of the layers deposited on the grating of period Λ) dictate the spectral position of the resonance wavelength of the DFB, λ_0_ (see eq 1). We previously demonstrated that the air/PMMA/FASnI_3_/glass structure is a suitable planar waveguide, where the high refractive index contrast between the FASnI_3_ (*n* ≈ 2.38 at λ=880 nm) and the surrounding layers, PMMA (*n* ≈ 1.48) and glass (*n* ≈ 1.5), results on propagating modes tightly confined in the semiconductor^19^ (see Figure S3, Supporting Information). In this way, *n*_eff_ mainly depends on the thickness of the FASnI_3_ (*d*_2_), so the degrees of freedom to design the device are reduced to d_2_ and Λ. Since the synthesis of our FASnI_3_ film is optimized to *d*_2_ = 200 nm, the HL method allows a straightforward tuning of Λ. In particular, we studied samples with Λ varying between 420 and 460 nm (see Table S1, Supporting Information), which allows one to sweep λ_0_ over a 40 nm bandwidth. The PL of the FASnI_3_ films used in this work (brown line in Figure S4 in Supporting Information) presents a Gaussian shape centered at λ = 860 nm with a full width at half-maximum (FWHM) of 80 nm; the 20–30 nm blue shift of the PL band obtained here compared to that reported previously^18,19^ is attributed to the corrugation imposed by the DCG film, which induces tensions in the semiconductor film. Here, it is important to consider the overlap of the absorption spectra with the PL (see the blue line in Figure S4, Supporting Information), which determines the optical gain at each emission wavelength, thus resulting on a red-shifted ASE band,^39^ located at 870–890 nm (see the red-shaded area in Figure 2a,b). To obtain an accurate prediction of λ_0_, the reflectivity of the device is simulated in transverse electric (TE) and transverse magnetic (TM) polarizations by a coupled-wave analysis combined with a multilayer algorithm.^40^ Simulations are optimized with *d*_1_ = 100 nm, *d*_2_ = 200 nm, and *d*_3_ = 500 nm and the refractive indices of the PMMA, fused silica, and FASnI_3_ obtained from the dispersion’s relationships presented elsewhere.^19,41^ To facilitate the simulations, the sinusoidal shape of the diffraction grating is approximated by a square shape with a given filling factor that accounts for the volume of the grating with respect to the total volume of the film. Since the sinusoidal shape of the grating only occupies a small region of the film (see Figure 1a), a filling factor of 0.1 is chosen to perform the simulations. At these conditions, the algorithm reasonably reproduces the experimental PL collected from samples with different periods. Indeed, according to these calculations, the TE_0_ mode is swept across the λ_0_ = 860–910 nm (see Figure 2a,b), overlapping the PL maximum at Λ = 430 nm and the ASE maximum at Λ = 440 nm. In TM polarization, the resonant mode arises at wavelengths shorter than 840 nm due to their smaller *n*_eff_,^19^ and only the tails of the modes overlap the emission bands. Interestingly, the absorption profile in FASnI_3_ (the blue curve in Figure S4, Supporting Information) significantly influences the shape of the resonant modes. The absorption coefficient broadens the resonance line width, limiting the quality factor to *Q* ≈ 10 (see Figure 2a), while in a lossless medium, the resonance narrows substantially, achieving *Q* = 170 (see Figure 2b). The first situation can be ascribed to PL below the threshold of the stimulated emission, where the transparence carrier density has not been achieved and the material does not present an optical gain. The second scenario corresponds to the transparency condition at the ASE threshold, where the photogenerated gain compensates for the absorption losses.

**Figure 2:**
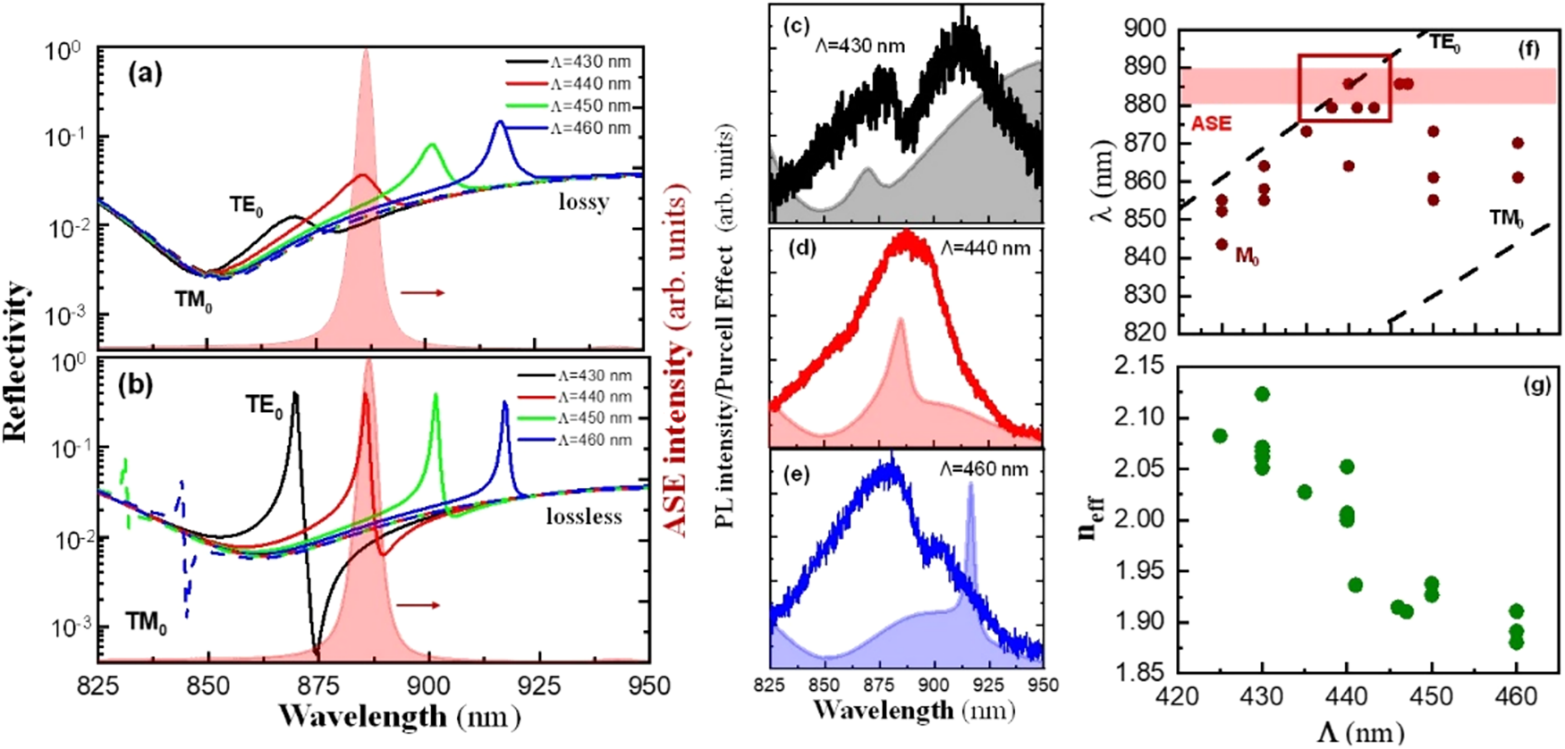
(a, b) Simulated reflectivity spectra for lossy case (below threshold) and lossless case (above threshold), respectively, in TE (solid line) and TM (dashed line) for the device geometry illustrated in Figure 1a for Λ = 430 nm (black), 440 nm (red), 450 nm (green), and 460 nm (blue). The ASE spectrum is shown in shaded red for reference. (c–e) Spontaneous emission spectra, solid line, and Purcell factor of the TE_0_ mode, shadow area, for Λ = 430 nm (c), Λ = 440 nm (d), and Λ = 460 nm (e). (f) Spectral position of the mode (brown symbols) and ASE (red area) as a function of Λ. Dashed lines represent the theoretical TE modes. (g) Experimental effective refractive index as a function of Λ.

The fabricated devices were characterized in a backscattering geometry by illuminating the surface samples with nanosecond pulses (1 ns and 100 Hz of repetition rate) at 532 nm. The size of the excitation spot, 420 × 100 μm^2^, was much smaller than the size diffraction gratings, and the intensity was controlled with neutral density filters. Samples were kept at room temperature in a nitrogen chamber to ensure the stability of the FASnI_3_ film during measurements. Solid lines in Figure 2c–e represent the collected PL emission of three representative samples with Λ = 430, 440, and 460 nm, respectively, pumped below the ASE threshold (*P*_th_). At these excitation conditions, the spectrum corresponds to the spontaneously emitted PL filtered by the shape of the TE_0_ resonance.^23^ In addition, the emission can be improved at specific wavelengths by the Purcell factor, which is estimated here as the product between the PL measured in a backscattering geometry (see Figure S4) and the spectral shape of the TE_0_ mode calculated in Figure 2a.^20^ In DFBs with TE_0_ at λ_0_ = 870 nm (Λ = 430 nm), the mode is broad by the significant absorption losses at this wavelength and the emission spectrum contains two Gaussian contributions (solid line in Figure 2c), the one observed at shorter wavelengths (875 nm) nicely agreeing with the peak of the estimated Purcell factor of the device (the gray shadowed area in Figure 2c). In the samples with Λ = 440 nm, the λ_0_ = 890 nm overlaps the ASE band well, representing an ideal condition for lasing.^27–31^ Indeed, the spontaneous emission spectrum measured for this sample shows a near-Gaussian shape centered at 880 nm (red solid line in Figure 2d), which is also consistent with the maximum Purcell factor (red shadowed area in Figure 2d). For longer grating periods (Λ = 450–460 nm), the spectral position of the TE_0_ mode is further red-shifted at wavelengths longer than 900 nm, resulting in an expected enhancement at this spectral region (see the green shadowed area in Figure 2e for Λ = 460 nm and Figure S5 for Λ = 450 nm). Nevertheless, the emission decoupled for this sample is located at 850 nm (green continuous line in Figure 2e), indicating that the collected signal corresponds to scattered PL, probably because of the poor overlap of the Purcell enhancement resonance with the emission band. In fact, except for the latter case, there is a correlation between the experimental values obtained for the PL peak (M0) wavelength and Λ for all gratings in the range Λ = 425–445 nm, as represented by the brown symbols in Figure 2f. Indeed, the experimental data obtained for M0 follow the predicted TE_0_ resonances (see the top dashed line in Figure 2f). Particularly, for Λ ≈ 440 nm, the mode overlaps the ASE band (shadowed area in red), which represents the tentative condition to obtain lasing.^27–31^ Finally, Figure 2g plots the effective refractive index, *n*_eff_, of the optical mode of the DFB structures as a function of Λ, as obtained from eq 1. The smaller *n*_eff_ compared to that calculated in a PMMA/FASnI_3_/glass waveguide structure (*n*_eff_ ≈ 2.1) is explained by the fact that the DFB FASnI_3_ film contains the corrugated grating inside, which has a refractive index lower than that of the semiconductor.

According to the results presented in Figure 2, the spectral overlap of the ASE band with λ_0_ will dictate the behavior of the device. Figure 3a–c plots the evolution of the decoupled PL with the excitation fluence (*P*) in the three representative samples shown in Figure 2c–e (Λ = 430, 440, and 460, respectively). All samples present two different regions. For *P* < *P*_th_, the collected PL is filtered by the mode of the cavity and grows linearly with *P*, as expected for the spontaneously emitted light. Nevertheless, for *P* > *P*_th_, the spectrum of the FASnI_3_ is characterized by a superlinear growth of a narrow, FWHM ≈ 10 nm, ASE band, centered at 880 nm. Moreover, the FASnI_3_ semiconductor proves to be an exceptionally efficient material for the formation of narrow (*Q* > 10^3^ at room temperature) and stable RL lines modulating the ASE band.^18,19^ At these conditions, the formation of RL on the surface of the film competes with the mode of the cavity,^31^ with the spectral position of M0 critically influencing the spectrum above threshold.^23^

**Figure 3:**
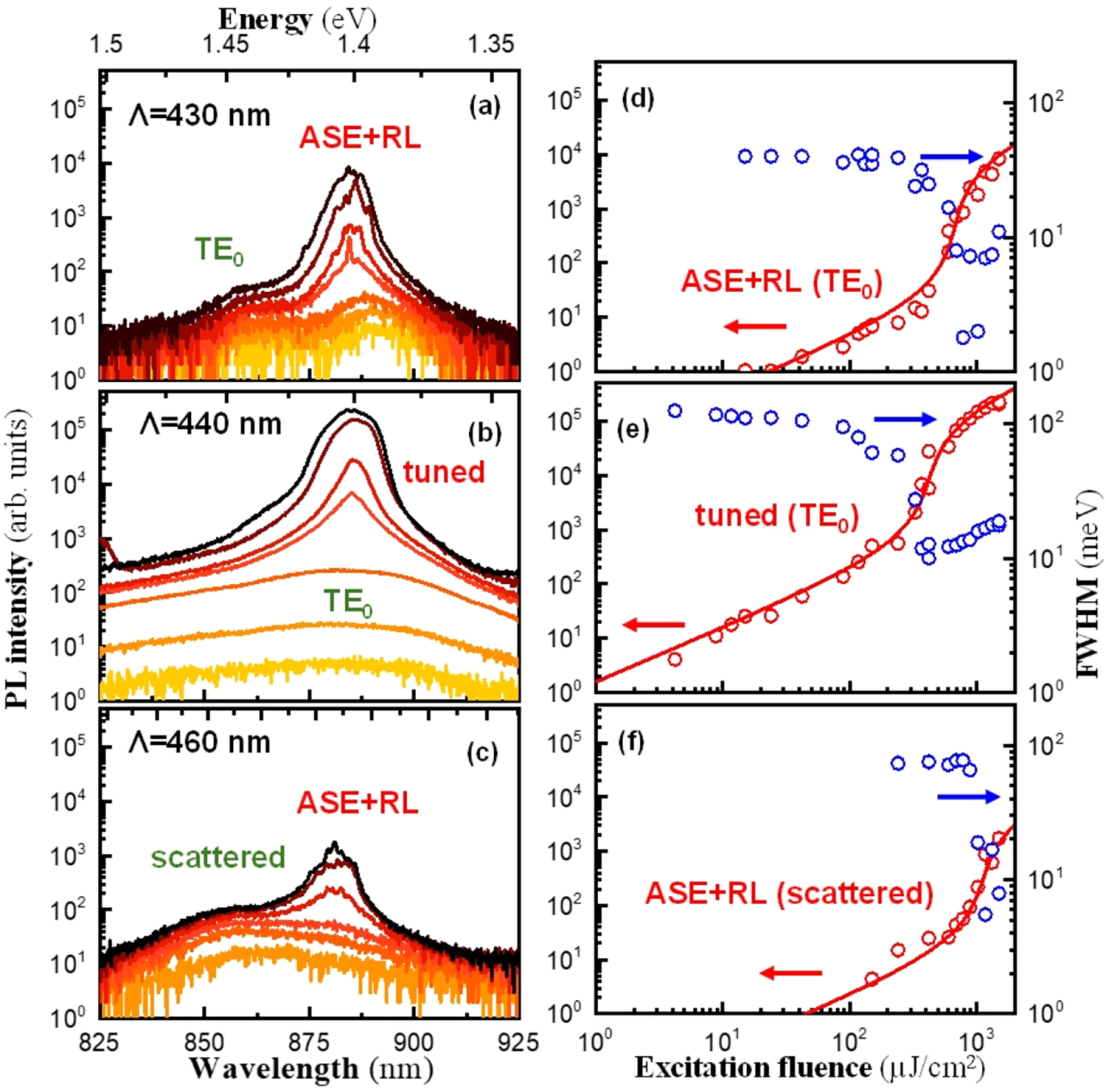
Dependence on the ASE/lasing in three representative devices with (a) Λ = 430 nm (extraction of the ASE and RL coupled to the TE_0_ mode), (b) 440 nm (DFB device), and (c) 460 nm (scattered ASE and RL). Log–log plot of the experimental PL intensity (red symbols) and fwhm (blue symbols) for (d) Λ = 430 nm, (e) 440 nm, and (f) 460 nm. Solid lines represent the fitting with the laser equations.

For Λ = 430 nm, the TE_0_ resonance, located at λ_0_ = 860 nm, is detuned with respect to the ASE band, and the device does not lase from the resonance. Nevertheless, at this spectral position, M0 overlaps the exciton in the absorption band edge, where the gain of the FASnI_3_ is maximum, resulting in an important Purcell effect and an efficient outcoupling of the RL lines (see Figure 3a). For Λ = 440 nm (Figure 3b), the resonance overlaps the ASE band, and the device behaves as an optical cavity where the TE_0_ extracted is dominant over the RL lines, which now are not observed in the extracted emission/lasing spectrum. Indeed, the peak intensity is maximum, about 1-fold higher than that obtained for the detuned DFB devices measured under the same excitation conditions. On the other hand, the line width of the emission/lasing spectrum above *P*_th_ decreases down to 6 nm, which indicates an experimental *Q* factor of 146, close to the *Q* obtained in the simulations in the lossless case (Figure 2b), where the transparence condition (gain = losses) is considered. This relatively low *Q* value of the devices prevented obtaining a line width below 1 nm, as typically observed for DFB lasers, which is ascribed here mainly to the small refractive index contrast between the corrugated grating (*n* = 1.55) and the FASnI_3_ (*n* = 2.38) together with losses in the polycrystalline grains and dispersion in the grating period on the fabricated devices. For Λ = 460 nm (Figure 3c) or Λ = 450 nm (see Figure S6), the resonance is red-shifted versus the ASE band in a spectral region where the semiconductor does not present an optical gain. In this way, only the tail of the TE_0_ mode only overlaps the ASE, resulting in a poor extracted light, about 2-fold smaller compared to the tuned device, which is attributed to the scattered light. Indeed, the spectrum decoupled from this grating is close to that obtained from the sample without grating; see Figure S7 (Supporting Information). Figure 3d–f presents the log–log plot of the PL intensity (*I*_PL_), open red symbols, and fwhm, open blue symbols, as a function of the incident laser fluence, *P*, for the three samples. The *I*_PL_–*P* dependence shows the *S*-curve characteristic of lasers that can be fitted with the following rate equation in the stationary state^42^

2 $$\frac{\partial N}{\partial t}=G-A_{\mathrm{nr}}\cdot N-A_{r}\cdot N-\Gamma \cdot \sigma \cdot \frac{c}{n_{\mathrm{eff}}}\cdot \left(n-N\right)\cdot S$$

3 $$\frac{dS}{dt}=\left(\Gamma \cdot \frac{c}{n_{eff}}\cdot \left(N-N_{0}\right)-\frac{1}{\tau_{c}}\right)\cdot S+A_{r}\cdot \beta \cdot N$$

where *N* is the density of carriers, *S* is the photon density, *A*_r_ is the radiative recombination rate (inverse of the recombination time), *A*_nr_ is the nonradiative recombination rate, σ is the gain cross section, *N*_0_ is the transparence carrier density, *n*_eff_ is the effective refractive index of the TE_0_ mode, Γ is the confinement factor of the mode in the FASnI_3_ film, β is the spontaneous emission factor, *c* is the speed of light, *τ*_c_ is the loss of photons inside the cavity, and *G* is the photogeneration of electron–hole pairs given by $G=\frac{P}{h\nu_{p}}\cdot \alpha_{p}$ , where *hv*_p_ and α_p_ are the photon energy and the absorption losses at the pump (532 nm), respectively. The parameters *A*_r_, *A*_nr_, *n*_eff_, and Γ are fixed to *A*_r_ = 0.7, *A*_nr_ = 0.2, *n*_eff_ ≈ 2, and Γ = 0.8 according to the mode analysis shown in Figure 2 and the experimental results obtained elsewhere.^19,23^

Following eqs 2 and 3, *N*_0_, σ, τ_c_, and β can be obtained from the best fitting to the experimental data. Particularly, a reasonable fitting is obtained for all samples with σ = 10^–19^ cm^2^, τ_c_ = 0.003 ns^–1^, and different values for *P*_th_ and β for all analyzed samples, as summarized graphically in Figure 4a,b, respectively. *P*_th_ is the excitation fluence needed to reach the carrier transparency condition, N_0_, of the FASnI_3_.^43^ The film without the DFB grating, excited under a similar backscattering geometry exhibits a *P*_th_ = 500–1000 μJ/cm^2^ (see Figure S4b), which is consistent with the threshold reported for a single FASnI_3_ film at room temperature.^18^ Nevertheless, an adequate optical architecture can provide a significant enhancement of the light emitted by the Purcell effect, which consequently reduces the ASE threshold.^21^ In the cavities with Λ = 425–435 nm studied here, *P*_th_ is reduced to 150–450 μJ/cm^2^ because the corresponding modes overlap the high energy side of the PL spectra, where the semiconductor presents a higher optical gain. In the resonant devices (Λ = 440–445 nm), we obtain a more important dispersion in the *P*_th_ data, *P*_th_ = 231–1000 μJ/cm^2^, that can be attributed to a different quality in the samples and a slightly smaller optical gain. Finally, the poor overlap of TE_0_ mode with the ASE band in the cavities with longer periods (Λ = 450–460 nm) results on the same high thresholds, *P*_th_ = 500–1000 μJ/cm^2^, obtained under backscattering geometry.

**Figure 4:**
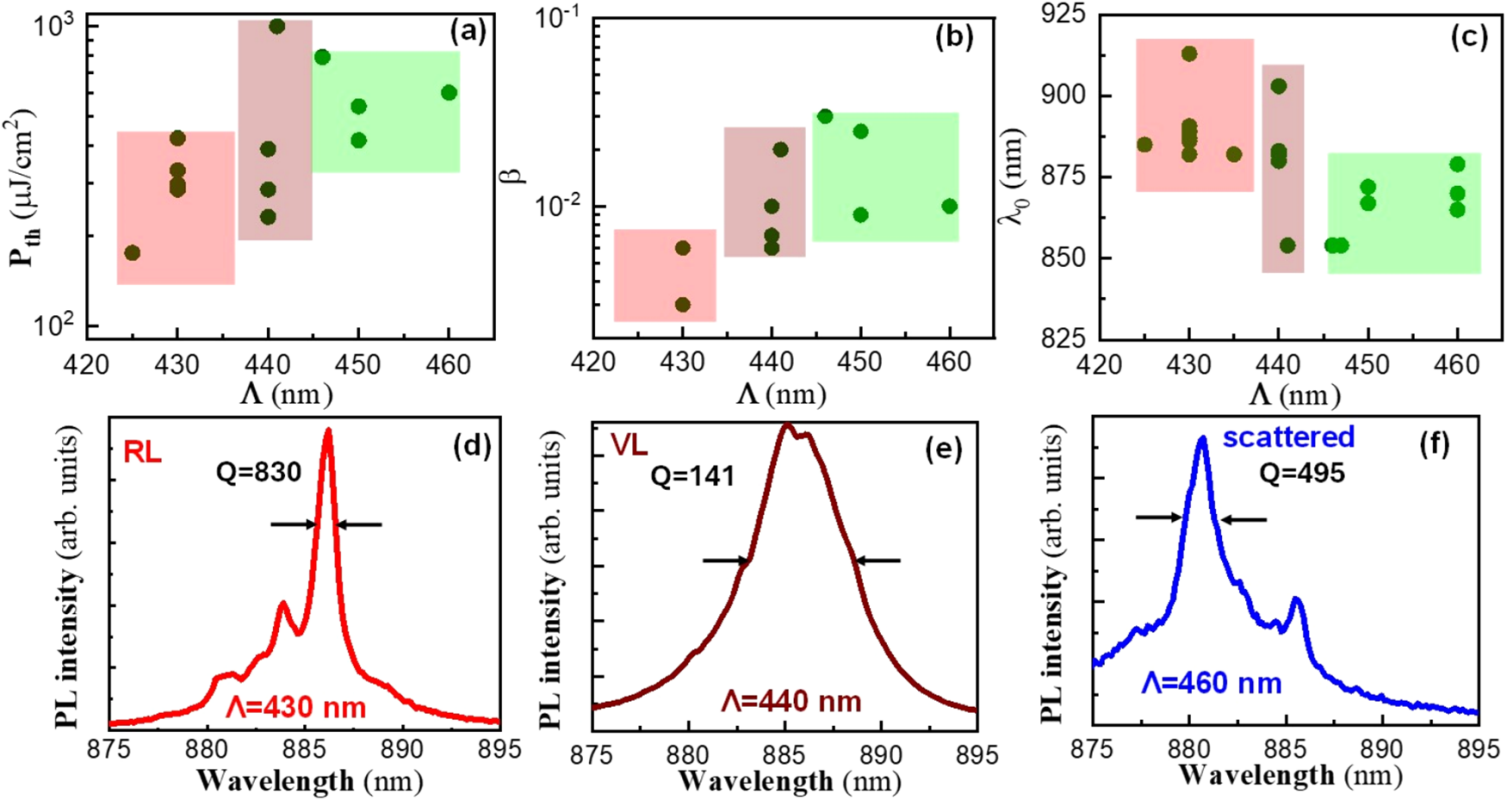
Dependence of the quality parameters of the devices as a function of Λ. (a) Threshold (*P*_th_), (b) spontaneous emission factor (β), and (c) emission wavelength (λ_0_). Representative spectra in the linear scale of devices with (d) Λ = 430 nm (extraction of the RL coupled to the TE_0_ mode), (e) Λ = 440 nm (enhanced ASE), and (f) Λ = 460 nm (scattered light); 880.

It is also interesting to compare the influence of Λ on the spontaneous emission factor, β; see Figure 4b. This factor indicates the percentage of emission coupled to a given optical mode. In a device producing RL, the factor is typically very low, here found to be β ≈ 0.003–0.006 for the untuned device, because the stimulated emission is distributed between several modes.^44^ Again, this figure of merit is similar to values found in RL observed under backscattering geometry.^18^ Contrarily, β can be significantly higher in a single-mode device that filters one RL peak^23^ or provides a certain directionality for emitted lasing light.^19^ In fact, for our DFB with Λ = 440 nm, β reaches higher values, β ≈ 0.06–0.02 (Figure 4b), because a tuned device facilitates the formation of the dominant mode (TE_0_ resonance) in the system. For scattered light, Λ = 445–460, β presents slightly higher values, 0.01–0.03 nm, because this scattered light mainly comes from the mode propagating along the waveguide plane, which seems to increase the spontaneous emission factor by directionality and light confinement.^19^ Finally, the shape and spectral position of the lasing spectra can be strongly controlled by tuning Λ; see Figure 4c. Figure 4e,f shows the spectra in linear scale for Λ = 430 nm, Λ = 440 nm, and Λ = 460 nm, respectively. In the untuned devices, the spectrum is composed by 2–3 narrow RL lines (1 nm) overlapping the tail of the TE_0_ mode. Here, the *Q* depends on the scattering resonance associated with long optical paths resulting on high *Q* = 495–830, in agreement with our previous results.^18^ In the tuned device, the spectrum corresponds to a single line at 885 nm with *Q* = 141, close to the value obtained in the calculations without losses.

## Conclusions

This work successfully demonstrates the control of the ASE and RL emitted by FASnI_3_ polycrystalline thin films through a polymeric grating. The gratings are fabricated in DCG through a low-cost holographic lithography (HL) technique, which enables the straightforward adjustment of Λ. Here, Λ is used as a tuning parameter to sweep the resonant wavelength (λ_0_) across the photoluminescence (PL) band of the semiconductor. When λ_0_ is optimally aligned with the ASE band, the device presents the optimum conditions for lasing, limited primarily by the modest *Q* of the cavity, around ≈100, which agrees with the theoretical predictions. Achieving a higher *Q* requires enhancing the grating effect, either by coating it with a high-index material or by positioning it on top, which nowadays presents risky degradation due to the instability of Sn in oxygen moisture or post-treatments. Additionally, an untuned device efficiently extracts the random lasing (RL) generated within the film. These findings hold significant promise for the Sn-perovskite community, paving the way for the development of cost-effective optical sources.

## Experimental Section

### Grating Fabrication

Surface-relief DCG gratings were fabricated in four steps^37,38^ (see Figure S6, Supporting Information): (1) DCG photoresist films of thickness 110 nm were deposited on FS substrates by spin coating a hot water solution at 40 °C of inert gelatin (Rousselot, 200 bloom) and ammonium dichromate used as a sensitizer. The concentration of gelatin was 2.2 wt % (with respect to water) and that of the sensitizer 35 wt % (with respect to gelatin); (2) films were exposed by HL in a Lloyd́s interferometer with light from a solid-state laser emitting at a wavelength of 460 nm. The period Λ of the interference pattern is given by Λ = λ/2 sin­(θ/2), where θ is the angle formed between the direction of the rays that produce the interference. During exposure, the absorbed light reduces Cr^6+^ ions to Cr^3+^ ions. In these places, chromic ions Cr^3+^ form cross-link bonds between carboxylate groups of neighboring gelatin molecules, which harden the gelatin. The average exposure, 1.5 mJ/cm^2^, is very high in order to produce a differential hardening between the exposed and unexposed areas as large as possible; (3) exposed plates were desensitized in a cold water bath (12 °C) for 6 s, followed by centrifugation at 500 rpm for rapid drying. The short time in this bath was found to be sufficient to remove the spectrophotometric signal corresponding to ammonium dichromate maintaining the uniformity of the film response; and (4) finally, surface-relief gratings were obtained by dry development in an oxygen plasma using a surface treatment machine Diener Zepto. The development degree was controlled by measuring the time evolution of the diffracted intensity.

### Materials

Tin­(II) iodide (SnI_2_, 99.99%), tin­(II) fluoride (SnF_2_, 99%), sodium borohydride (NaBH_4_, 96%), *N*,*N*-dimethylformamide (DMF, 99.8%), and dimethyl sulfoxide (DMSO, 99.8%) were purchased from Sigma-Aldrich. Formamidinium iodide (FAI. 99.99%) was purchased from Greatcell Solar Materials. These materials were used as received with no further purifications. Dipropylammonium iodide salt was synthesized with the following procedure: 10 g of dipropylamine was added to 30 mL of cold EtOH. Then, 13 mL of HI was added dropwise to the flask under vigorous stirring. The white solid formed after the addition of HI was filtered and washed with 100 mL of cold diethyl ether, and it was recrystallized using EtOH.

### Perovskite Solution Preparation

To prepare the FASnI_3_ precursor solution, 298 mg of SnI_2_ (0.8 M), 123.8 mg of FAI (0.72 M), 36.65 mg of DipI (0.16 M), 0.1 mg of sodium borohydride, NaBH_4_ (0.0026 M, this value might vary depending on the batch and purity of the precursor), and 12.48 mg of SnF_2_(0.08 M) were dissolved in 1 mL of a mix solution of DMSO/DMF (9:1, v/v) and stirred overnight at room temperature. The combination of NaBH_4_ and DipI additives produced a synergistic effect in FASnI_3_ films increasing their stability, where the former was a reducing agent avoiding Sn^2+^ oxidation while the latter acted as a passivating agent, resulting in an enhancement of their optoelectronic properties under N_2_ and ambient conditions, as discussed in previous works.

### FASnI_3_ Film Fabrication

The FASnI_3_ films were prepared in a N_2_-filled glovebox. The perovskite solution was deposited by spin coating at 4000 rpm and 4000 of acceleration for 50s. Then, during the spin, 400 μL of chlorobenzene was dropped on top of the substrate after 20 s of spinning; afterward, a two-step annealing was performed at 70 and 100 °C for 1 and 20 min, respectively. After the perovskite film was formed and cooled, a PMMA solution (100 mg/mL in CB) was spin-coated at 3000 rpm and 3000 of acceleration, and then the substrate was annealed at 100 °C for 3 min.

### Film Characterization

SEM images were taken with a field emission scanning electron microscope ((FEG-SEM)­JEOL 3100F) operated at 15 kV. The reflectivity/transmittance (absorption) of the samples was obtained by means of a commercial reflectometer (NanoCalc-2000 from Mikropack).

The PL of the FASnI_3_ was measured by pumping the films with 532 nm and collecting the backscattered PL into a fiber optics connected to a commercial HR4000 Ocean Optics spectrograph.

XRD patterns of the films were measured using an X-ray diffractometer (D8 Advance, Bruker-AXS) (Cu Kα, wavelength λ = 1.5406 Å) with a Bragg angle range of 4–70 ° and a step size of 0.05°.

To characterize the photoluminescence and ASE, the devices were pumped at a 532 nm pulsed laser (1 ns, 100 Hz to 20 kHz) from the top of the samples with a concave mirror that focused the beam on 420 × 100 μm^2^ area. The excitation fluence was controlled with neutral density filters. The emitted PL was collected from the top of the sample with a 20× microscope objective mounted on a XYZ stage. After a 550 nm long-pass filter, the collected PL is dispersed by a grating spectrograph (DNS-300 from DeltaNu) and detected by a back-illuminated Si CCD (DV420A-OE from Andor) at its exit. In some samples, the PL is analyzed by a grating spectrograph (Kimera 328i from Andor) and detected by a back-illuminated S-CMOS CCD (Marana-6 from Andor) at its exit. During the characterization, all samples were kept on an N2 chamber to ensure stability.

## Supplementary Material



## Notes

List of samples; structural characterization of the FASnI_3_ films; mode profile; absorption and PL; PL, ASE, and RL with Λ = 450 nm; ASE without the DFB structure; and grating fabrication (PDF)
