# Supplementary material for: Control of Stimulated Emission of Tin Perovskites through Polymeric Diffractive Gratings
Source: ACS Photonics. 2025 May 21;12(6):3154–62. doi: 10.1021/acsphotonics.5c00471 (PMC12183764; doi:10.1021/acsphotonics.5c00471)
Supplement: Supplementary file 1 [file ph5c00471_si_001.pdf]

Control of stimulated emission of tin perovskites through polymeric diffractive gratings

Juan P. Martínez-Pastor, [1] Jesús Sánchez-Díaz, [2] José M. Villalvilla,[3] Sandra Soriano-Díaz,[1,4] José A. Quintana,[5] Iván Mora-Seró, [2*] María A. Díaz-García[3*] Isaac Suárez, [1,4*]
[1] *UMDO, Instituto de Ciencia de los Materiales, Universidad de Valencia, Valencia 46980, Spain*
[2] *Institute of Advanced Materials (INAM), Universitat Jaume I, Castelló de la Plana, Castelló 12006, Spain*
[3] *Departamento de Física Aplicada and Instituto Universitario de Materiales de Alicante (IUMA), Universidad de Alicante, Alicante 03080, Spain*
[4] *Departamento de Ingeniería Electrónica, Escuela Técnica Superior de Ingeniería, Universidad de Valencia, Valencia 46100, Spain*
[5] *Departamento de Óptica, Farmacología y Anatomía and IUMA, Universidad de Alicante, Alicante 03080, Spain*

S1. List of samples.

Table S1. Summary of samples (Λ: grating period; M0: experimental emission peak)

Sample	Λ (nm)	M0 (nm)
1	430	858-864
2	440	864
3	450	855-861
4	460	861
5	430	864
6	440	885
7	460	870
8	425	852
9	435	873
10	425	843-855
11	430	855
12	441	879
13	446	885
14	447	885
15	443	879
16	438	879

S2. Structural characterization of the $FASnI_3$ films.

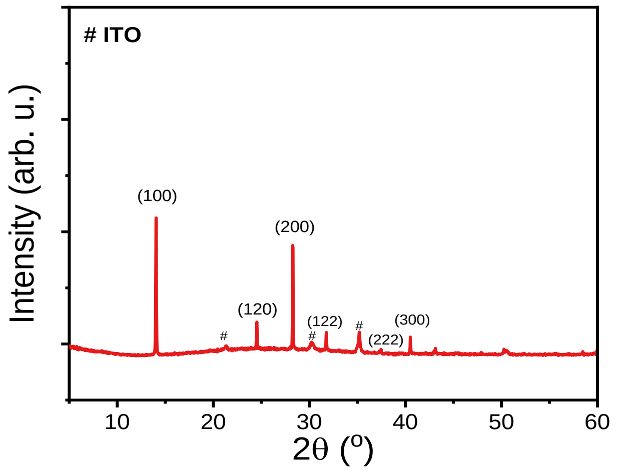

Figure S1. XRD patterns.

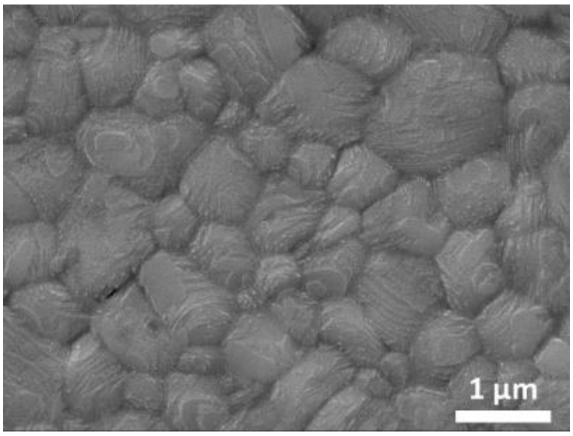

Figure S2. Top SEM image.

S3. Mode profile.

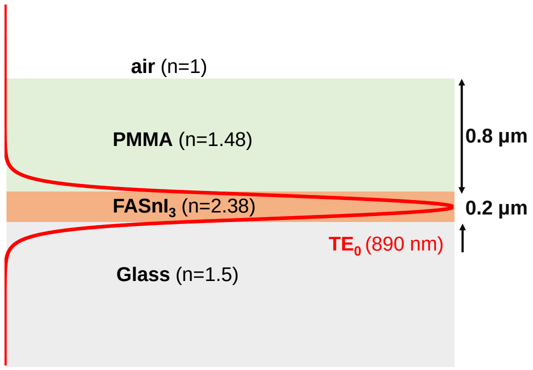

Figure S3. Simulation of the TE_0 mode confined on a $FASnI_3$ thin film.

S4. Absorption and PL.

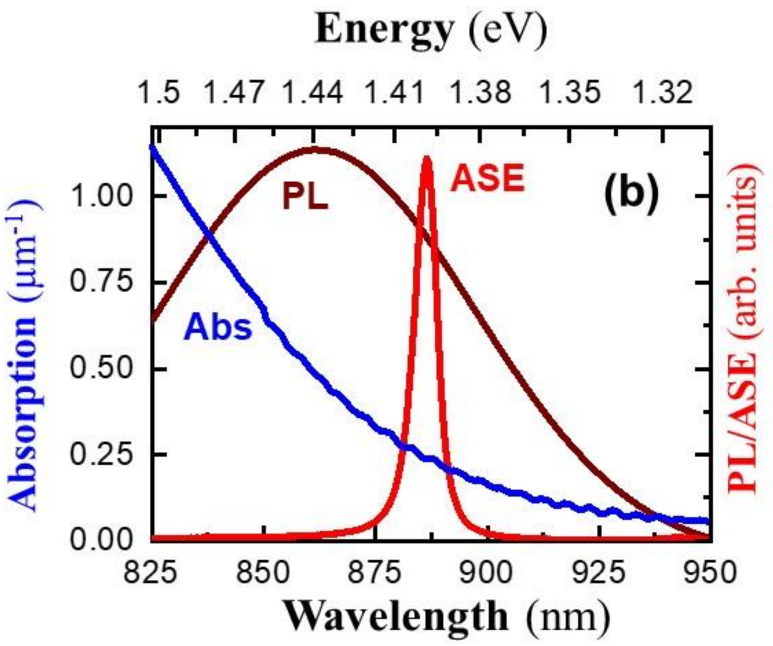

Figure S4. Absorption coefficient (blue), PL of FASnI3 (brown) and ASE spectra (red).

S5. PL, ASE and RL with Λ=450 nm.

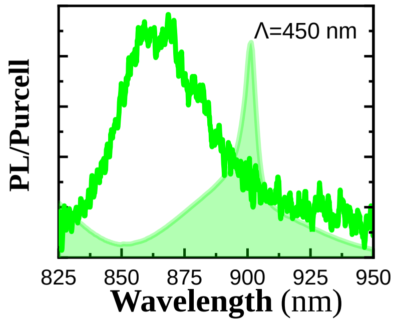

Figure S5. Spontaneous emission spectra, solid line, and Purcell factor of the TE0 mode for Λ=450 nm.

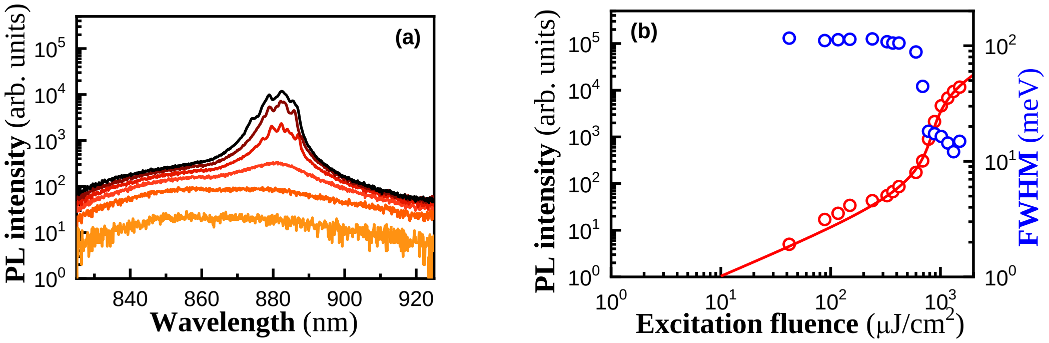

Figure S6. Results with Λ=450 nm. (a) Spectra for different excitation fluences. (b) Log-log plot of the experimental PL intensity (red symbols) and FWHM (blue symbols).

S6. ASE without DFB structure.

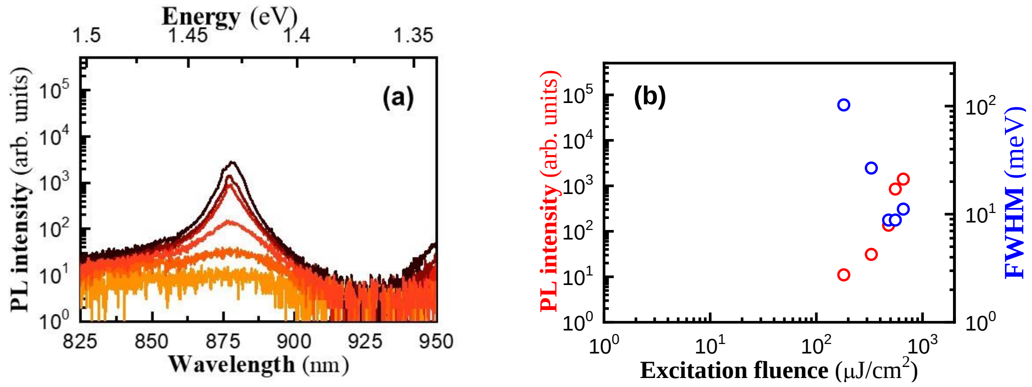

Figure S7. Dependence on the ASE in the $FASnI_3$ film (without grating) (a) Spectra for different excitation fluences. (b) Log-log plot of the experimental PL intensity (red symbols) and FWHM (blue symbols).

S7. Grating fabrication.

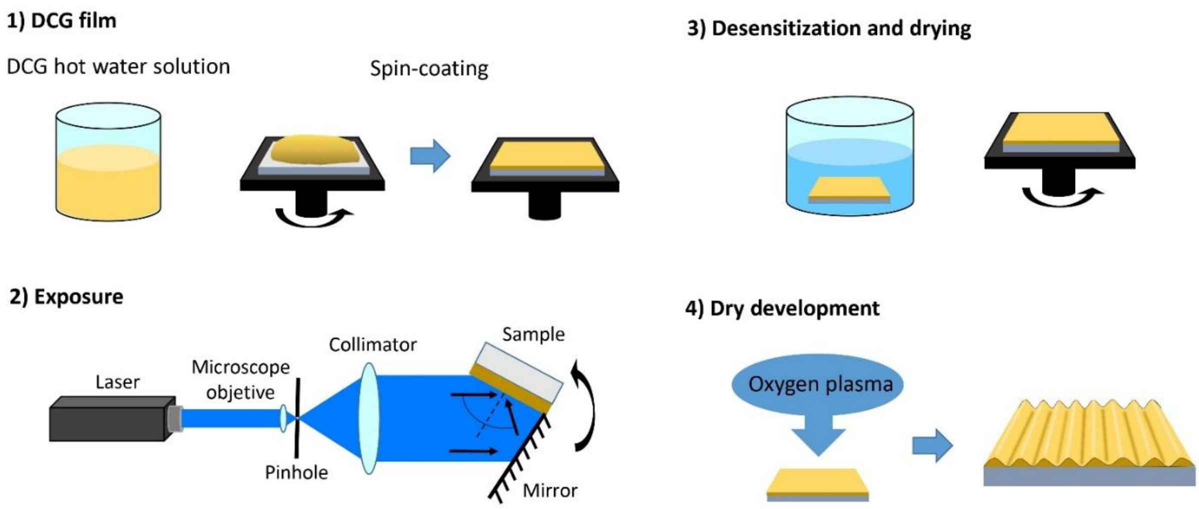

Figure S8. Steps for grating fabrication in dichromated gelatin (DCG): 1) A hot water solution of inert gelatin and ammonium dichromate is prepared and used to create a DCG photoresist film on a fused silica substrate through spin coating; 2) The DCG film is holographically exposed in a Lloyd's interferometer using coherent light; 3) The DCG film is desensitized in a water bath and dried via centrifugation; 4) An oxygen plasma treatment (dry development) is applied to achieve a surface relief grating.
